# Effect of Different Isothermal Time on Microstructure and Mechanical Property of the Low-Carbon Steel Treated by Dual-Stable C-Mn Partitioning Process

**DOI:** 10.1155/2020/5931721

**Published:** 2020-03-07

**Authors:** Cainian Jing, Xiaoyun Ding, Daomin Ye, Jingrui Zhao, Tao Lin, Shubo Xu

**Affiliations:** School of Materials Science and Engineering, Shandong Jianzhu University, Jinan 250101, China

## Abstract

The stability of retained austenite was improved by the dual-stable C-Mn partitioning process. The phase transformation and element diffusion of dual-stable C-Mn partitioning process of tested steel were investigated by means of EPMA, SEM, OM, tensile testing machine, and other analysis methods. The effects of the first and second austenite stabilization time on the microstructure and mechanical properties of low-C-Si-Mn steel were studied, respectively. The enrichment of C and Mn elements is obvious after the dual-stable C-Mn partitioning process, and the microstructure of the tested steel is constituted of martensite, ferrite, and retained austenite. Compared with the conventional Q&P steel, the tensile strength of the steel treated by the dual-stable C-Mn partitioning process is slightly lower, but the plasticity is improved significantly. The tensile strength is 875-910 MPa, the elongation is 20-24%, and the product of strength and elongation can reach 21 GPa·%.

## 1. Introduction

In recent years, with the continuous development of the automotive industry, resources and environmental issues have become increasingly prominent, which puts higher demands on the energy efficiency of automobiles. Automobile lightweight is an inevitable trend in the development of the automotive industry, and the application of high-strength steel is an effective technical mean for lightweight vehicles [1]. In 2003, Speer et al. [2] of the US State School of Mines proposed the Q&P (Quenching & Partitioning) process and established a thermodynamic model to study the problem of the C element partitioning of the experimental steel between the temperature of *M*_s_ and *M*_f_. In the following ten years, the third generation of high-strength steel represented by Q&P steel and medium manganese steel were rapidly developed [3, 4]. The room temperature microstructure of Q&P steel is martensite and retained austenite [5, 6]. Martensite as matrix ensures high strength, and the TRIP (Transformation Induced Plasticity) effect of retained austenite makes Q&P steel have good plasticity [7]. Studies have shown that the regulation of retained austenite is an extremely important factor among many factors affecting the mechanical properties of high-strength steel [5, 8]. The tested steel is heated to the two-phase zone for heat preservation, and the coordination of elements such as Mn and Cu can increase the stability of austenite to a certain extent, and at the same time, a part of ferrite can be introduced to further improve the plasticity to obtain the comprehensive mechanics of the experimental steel [9, 10].

The dual-stable C-Mn partitioning process is to improve the stability of austenite by two austenite stabilization processes. First, annealing in the two-phase region promotes the partitioning of C and Mn elements from ferrite to austenite [11], that is, the high-temperature austenite stabilization process; the second process is the use of low-temperature carbon distribution similar to the traditional “one-step” Q&P process. During the second process, after annealing in the two-phase region, the experimental steel is quenched to a temperature between *M*_s_ and *M*_f_ to maintain the C element partitioning from martensite to austenite [12]. In this study, the low-carbon-silicon-manganese steel was selected as the raw material. The low-carbon steel distribution effect was improved by two austenite stabilization processes, and the problem of low-residual austenite amount and poor plasticity after quenching was solved. The effect of different austenite stabilization time on the microstructure and mechanical properties of the tested steel was studied.

## 2. Experimental Materials and Methods

**Figure 1:**
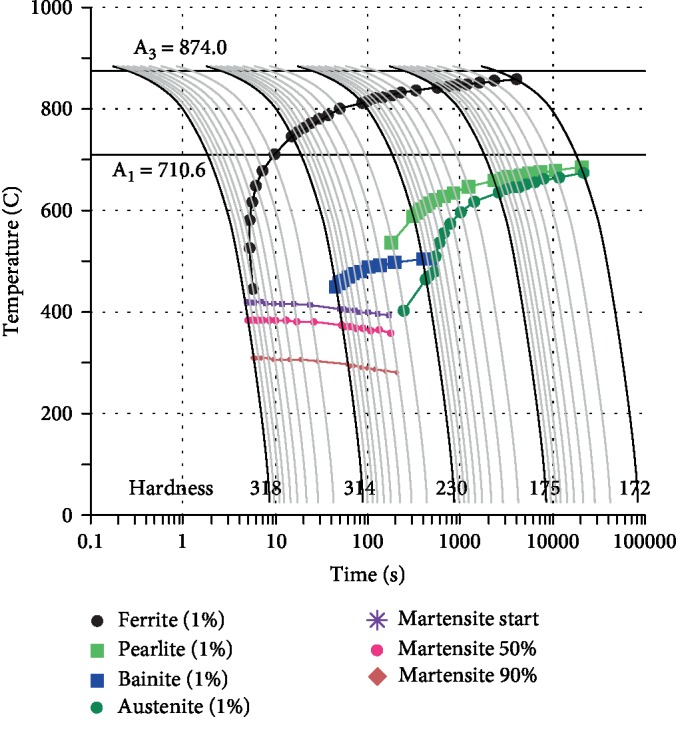
The CCT diagram of tested steel.

The material used in the test was a cold-rolled low-carbon-silicon-manganese steel having a thickness of 1.5 mm, and a standard tensile specimen having a gauge length of 25 mm was prepared by wire cutting according to the ASTM E8 standard. The initial structure of the tested steel consists of ferrite and pearlite. The main chemical composition thereof is shown in Table 1. The phase transition point of the tested steel was calculated by the empirical formula and JMatPro 7.0 software: 710°C for the austenite transformation starting temperature (*A*_c1_), 874°C for the austenite transformation end temperature (*A*_c3_), and 410°C for the martensite transformation starting temperature (*M*_s_).  Figure 1 is the CCT diagram of tested steel. The traditional Q&P process and dual-stable C-Mn partitioning process are applied to the experimental steel to study the influence of process parameters on microstructure and mechanical properties.

**Figure 2:**
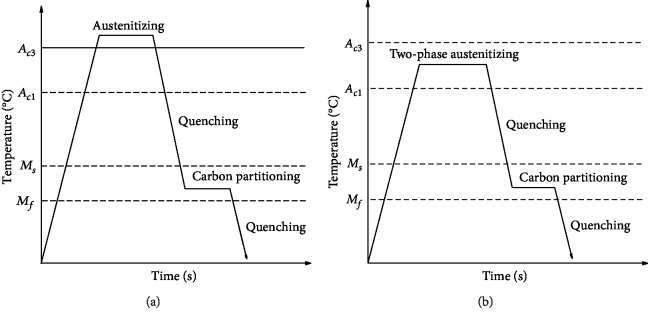
Schematic of heat treatment: (a) Q&P process and (b) dual-stable C-Mn partitioning process.

Figure 2 illustrates the Q&P process and the dual-stable C-Mn partitioning process. The heating process was carried out in the vacuum heat treatment furnace. The dual-stable C-Mn partitioning process was to rapidly heat the tested steel at 10°C/min to the two-phase region of ferrite and austenite at 820°C for 3 min, 5 min, 7 min, and 9 min, respectively, for the first austenite stabilization process. Then, the tested steel was quenched to 240°C, holding for 10 sec, 20 sec, 30 sec, 40 sec, and 50 sec, respectively, for the second stabilization process of austenite. Finally, the sample steel was quenched with water to room temperature. As the control group, the experimental steel was kept at 930°C for 5 min to make sure the completion of austenitic transformation, then quenched to 240°C for carbon distribution, and finally quenched to room temperature with water.

## 3. Results and Analysis

### 3.1. Effect of Dual-Stable C-Mn Partitioning Process on Microstructure of Tested Steel

#### 3.1.1. Effect of Dual-Stable C-Mn Partitioning Process on Elemental Partitioning Behavior

**Figure 3:**
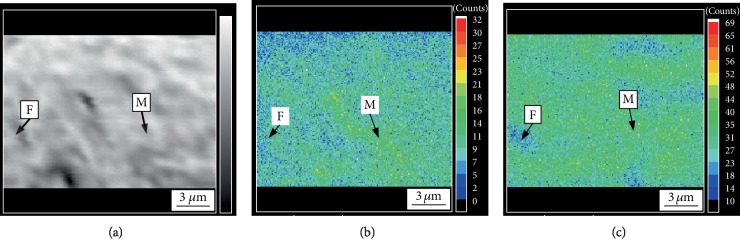
(a) The microstructure image, the EPMA images of (b) C, and (c) Mn element distribution of tested steel after Q&P process.

**Figure 4:**
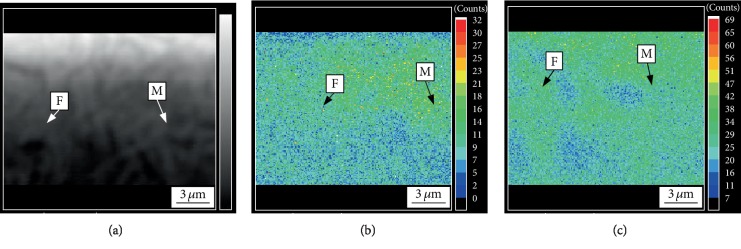
(a) The microstructure image, the EPMA images of (b) C, and (c) Mn element distribution of tested steel at 820°C for 5 min and then at 240°C for 30 s.

Electron microprobe analysis (EPMA) was performed on the tested steel after the dual-stable C-Mn partitioning process and the traditional Q&P process, respectively.  Figure 3 shows the distribution of C and Mn elements in the tested steel after treatment of the traditional Q&P process.  Figure 4 shows the distribution of C and Mn elements after the dual-stable C-Mn partitioning process. Comparing Figures 3 and 4, C and Mn elements are obviously enriched between the lath of martensite in both the two processes. However, the enrichment of the elements in the dual-stable C-Mn partitioning process is more obvious. It can be seen from Figures 3 and 4 that the distribution position of C element is basically consistent with that of Mn element. The reason is that the distribution process of Mn element causes the originally distributed Mn atoms to migrate and generate after obtaining sufficient diffusion activation energy. Because the radius of Mn atom is slightly larger than that of iron atom and much larger than that of carbon atom, the migration of Mn causes lattice distortion. As an interstitial atom, C atom is more easily retained in the lattice gap caused by the migration of Mn atoms, resulting in the accumulation of more C atoms near the Mn atoms and the phenomenon of the same distribution position of Mn and C. In this process, the Mn content of austenite also decreased gradually. In this process, the martensite transformed from austenite after quenching retains the distribution characteristics of Mn elements in austenite, i.e., the Mn content in the central region is higher than that in the surrounding area [13]. The C element partition after quenching between *M*_s_ and *M*_f_ can stabilize the retention of more retained austenite to room temperature [14]. Compared with the traditional Q&P process, the dual-stable C-Mn partitioning process improves the austenite stability by the two austenite stabilization processes of C, Mn elemental partitioning at high temperature, and C elemental partitioning at low temperature. The tested steel has both sufficient strength and excellent plasticity so that it has good comprehensive mechanical properties.

#### 3.1.2. Effect of the First Austenite Stabilization Time on Microstructure

**Figure 5:**
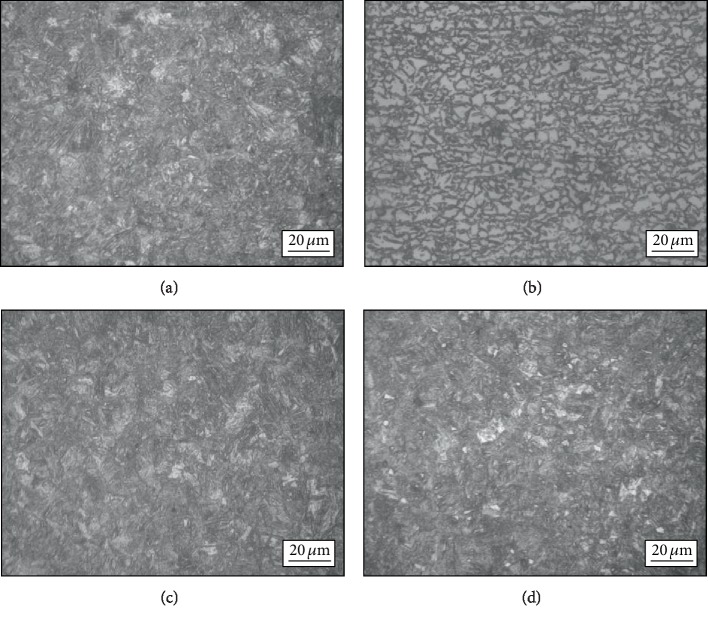
The OM micrographs of the first austenite stabilization for (a) 3 min, (b) 5 min, (c) 7 min, and (d) 9 min.

**Figure 6:**
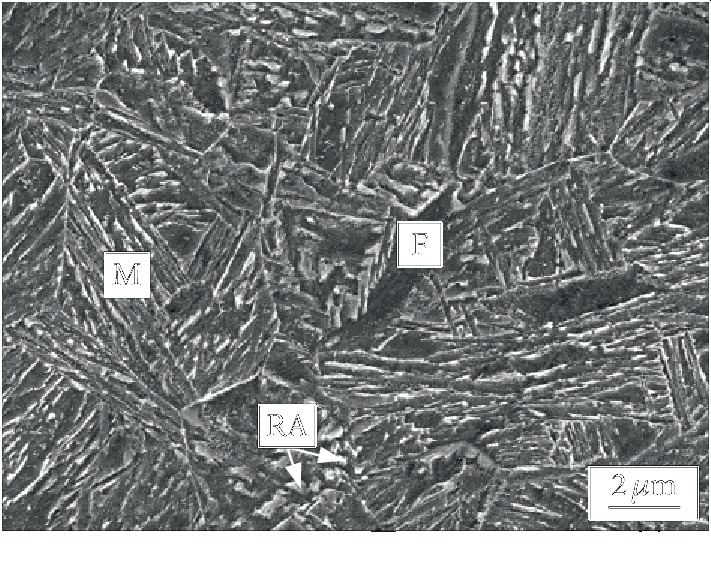
The SEM micrographs of tested steel of dual-stable C-Mn partitioning process test.

The effect of the first austenite stabilization time on the microstructure of the tested steel during the dual-stable C-Mn partitioning process was investigated. The metallographic structures of the tested steel at 820°C for different austenite stabilization time (3, 5, 7, and 9 min) then at 240°C for 30 s are shown in Figure 5. The metallographic structure of the tested steel is a composite structure of lath martensite and ferrite. With the extension of the first austenite stabilization time, the ferrite volume fraction first increases and then decreases, and the morphology also changes. The volume fraction of ferrite in the tested steel annealing at 820°C for 3 min is low. When the annealing time increases to 5 min, the ferrite is mainly in bulk shape and the volume fraction of ferrite increases, which may be due to the partial growth of ferrite grains with the increase of the first austenite stabilization time [15]. As the austenite stabilization time continues to prolong, the austenite interface gradually grows into the ferrite and the nearby ferrite is annexed, which results in a decrease in the ferrite volume fraction in the microstructure at room temperature.  Figure 6 is an SEM image of the dual-stable C-Mn partitioning process annealed at 820°C for 5 min. The microstructure of the tested steel is austenite, ferrite, and a small amount of retained austenite. The martensite is lath-like, and the slats of the martensite group have different beam positions.

#### 3.1.3. Effect of the Second Austenite Stabilization Time on Microstructure

**Figure 7:**
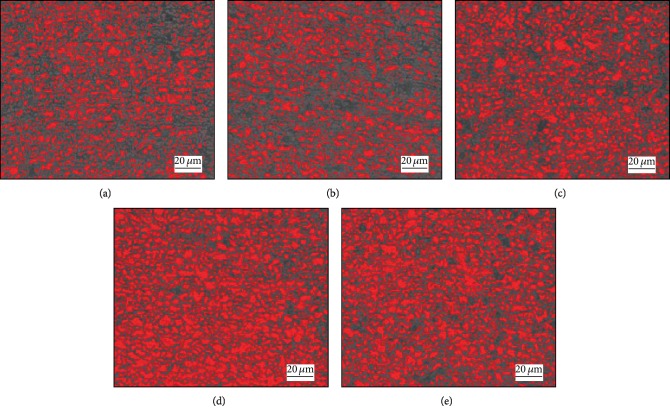
The morphology of martensite and ferrite (red) after partitioning for (a) 10 sec, (b) 20 sec, (c) 30 sec, (d) 40 sec, and (e) 50 sec.

In order to explore the influence of the second austenite stabilization time on the tested steel structure, the tested steel was quenched to 240°C (between the temperature of *M*_s_ and *M*_f_) and kept in this temperature for 10-60 sec after 5 min of first austenite stabilization process.  Figure 7 shows the metallographic structure of the experimental steel for different second austenite stabilization time, where the red parts represent ferrite. We calculated the fractions of ferrite by the Photoshop CC software, and the results are shown in Table 2. It was found that the microstructure of the dual-stable C-Mn partitioning steel consists of martensite, ferrite, and retained austenite from the SEM analysis. It can be seen from Figure 7 that a granular or flake-like ferrite structure is uniformly and distributed scatteredly in the lath martensite gap. When the second austenite stabilization time is 10 sec, the amount of martensite is relatively high and decreases gradually with the time prolonging. By analyzing the SEM photograph of the tested steel, it is known that the residual austenite is distributed in a film form at the boundary between the interlaced lath martensite and the bulk ferrite due to the low carbon content of the sample steel. When the steel plate is subjected to external force, the strip martensite of the plate provides high strength to resist the deformation of the steel plate. The TRIP effect of retained austenite impacted by external force induces martensite and retards necking, and the external impact work is absorbed with the relatively soft ferrite. The uniform distribution of the three phases makes the steel possess the comprehensive mechanical property of high strength and high plasticity.

### 3.2. Analysis of Phase Transition Process of Dual-Stable C-Mn Partitioning Process

**Figure 8:**
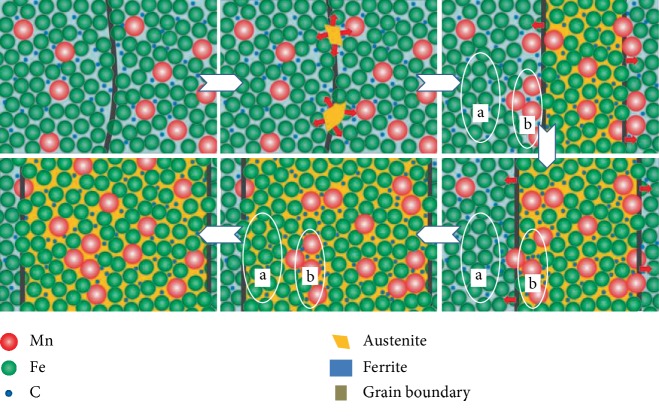
The schematic representation of the first austenite stabilization process.

According to the EPMA image of the microstructure and element distribution of the dual-stable C-Mn partitioning process, the hypothesis of the phase transformation process is proposed, and the first austenite stabilization process is analyzed in combination with Figure 8. The solid solubility of C and Mn in ferrite is low. When the first austenite stabilization process is maintained, the austenite begins to nucleate after reaching the phase transition point. The diffusion of C element from ferrite to austenite is completed first [16]. The diffusion rate of Mn element is higher in ferrite than in austenite. Mn undergoes short-range diffusion after obtaining activation energy. It migrates from ferrite to ferrite/austenite boundary and aggregates there. The Mn element is distributed at the ferrite/austenite interface, and its diffusion rate in the ferrite is much higher than that in the austenite, resulting in a difference in the concentration of Mn elements at the two sides of the interface. With the diffusion of Mn element, Mn-rich region b and Mn-depleted region a were formed at the grain boundary and central region of ferrite, respectively. The Mn content in the grain boundary is relatively high, and the austenite grains are small right now. Although this process is accompanied by the migration of austenite grain boundaries to ferrite, the grain boundary growth is not obvious since Mn has the effect of stabilizing austenite and inhibiting grain growth. With the increase of the first austenite stabilization time, the Mn atoms originally gathered at the boundary gradually migrate into the austenite, and the grain boundaries migrate to the ferrite side. As the Mn-poor ferrite area is gradually “engulfed” by the grain boundaries, the austenite grows gradually, and the content of Mn element in the transformed austenite gradually decreases. After quenching, martensite retains the distribution characteristics of Mn in austenite, and the Mn content in the central region is higher than that in the surrounding area. As the first austenite stabilization time increases, the grain size of austenite and ferrite changes, and the C and Mn elements uniformly diffuse in the austenite. With the increase of time, Mn element diffuses from the interface to the austenite grain. At the austenite grain boundary, the content of Mn is continuously decreasing, and the grain growth resistance decreases. As the grain grows, the internal average Mn content is decreased, and the first austenite stabilization effect is reduced. When the time further increases, the growth of austenite grain is dominant, the austenite grains become larger, the residual retained austenite content gradually decreases after quenching, and the martensite lath spacing becomes wider, which deteriorates the mechanical properties of the tested steel. Therefore, the first austenite stabilization time has an essential influence on the microstructure and mechanical properties of the tested steel.

**Figure 9:**
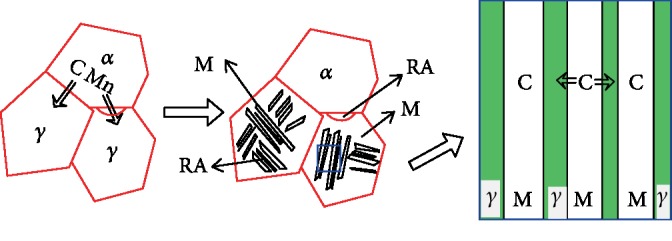
The schematic representation of the dual-stable C-Mn partitioning process.

A schematic diagram of the dual-stable C-Mn partitioning process is analyzed in conjunction with Figure 9. When the tested steel is heated to the temperature above the starting transformation temperature of austenite at a suitable heating rate and maintained, C and Mn elements are enriched in austenite, increasing the high-temperature stability of austenite [17]. After the first quenching, a composite structure of martensite, ferrite, and retained austenite is obtained [18]. The saturated solid solubility of C and Mn in ferrite is extremely low, and the first quenching temperature is 240°C. Therefore, the influence of C and Mn diffusion in ferrite can be ignored [19]. In the carbon distribution process, i.e., the second austenite stabilization process for the tested steel, similar to the traditional one-step Q&P process, C element diffuses from martensitic lath to the adjacent thin film austenite or sheet austenite, increasing the stability of austenite at room temperature [20]. At the same time, part of the ferrite increases the grain boundary density and prolongs the time of the dynamic equilibrium of the C element. The optimal second austenite stabilization time is extended to suppress the precipitation of carbides in this process. Finally, more retained austenite is obtained at room temperature, thereby improving the overall mechanical properties.

### 3.3. Influence of Dual-Stable C-Mn Partitioning Process on Mechanical Properties

#### 3.3.1. Effect of the First Austenite Stabilization Time on Mechanical Properties

**Figure 10:**
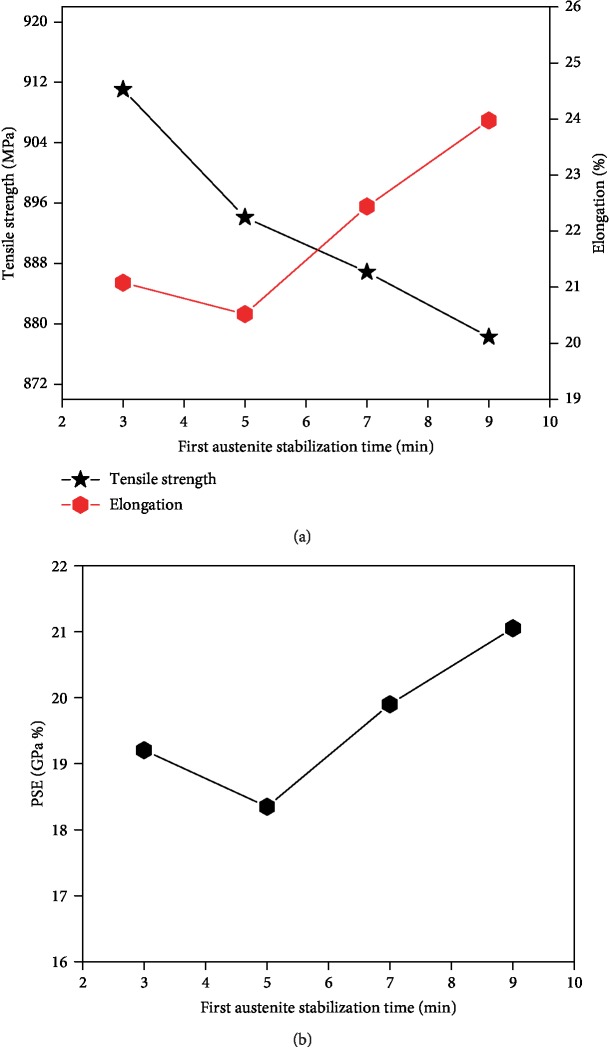
Mechanical properties of these steels after first austenite stabilization for 3 min, 5 min, 7 min, and 9 min: (a) strength and elongation and (b) product of strength and elongation.

In the first austenite stabilization process, the stability of austenite is increased by the distribution of C and Mn elements, which has an effect on the mechanical properties. The tested steel was incubated at an austempering temperature of 820°C for 3-9 min and then subjected to the same dual-stable C-Mn partitioning process. The tensile strength and elongation at break of the tested steel change with the first austenite stabilization time, as shown in Figure 10(a). The change of the product of strength and elongation with the first austenite stabilization is shown in Figure 10(b). With the increase of the first austenite stabilization holding time, the tensile strength gradually decreases, while the elongation after breaking first decreases and then increases and reaches a minimum value at 5 min. The change rule of the product of strength and elongation is basically the same as that of elongation after fracture. The product of strength and elongation of the tested steel showed a minimum at 5 min, and the comprehensive mechanical properties were relatively poor. After the dual-stable C-Mn partitioning process, the tensile strength of the tested steel is between 875 MPa and 910 MPa, and the elongation after fracture is up to 24%. The product of strength and elongation is between 18.3-21 GPa·%. Under the dual-stable C-Mn partitioning process, the mechanical properties may be changed. The tested steel is insulated in the two-phase zone, and the austenite gradually grows. When holding for 3 minutes, the austenite and ferrite grains are fine, which can be observed from the microstructure observation. According to the Holpeci formula, the tested steel has a good match of strength and plasticity due to the effect of fine-grain strengthening. With the prolonged holding time, the grain growth has a major influence on the mechanical properties of the tested steel [21]. In the range of 5-7 min, the nucleation rate of austenite increases, and the volume fraction of ferrite decreases. The first austenite stabilization is sufficient, and the C and Mn elements diffuse into the high-solubility austenite, which increases the high-temperature stability of austenite. In addition, the Mn element has an effect of hindering the growth of crystal grains, so that the grain boundary growth rate is relatively reduced after the austenite/ferrite interface is rich in Mn [22, 23]. More retained austenite is obtained during the dual-stable C-Mn partitioning process, which increases the elongation of the tested steel after fracture. However, the ferrite content continues to increase, resulting in a slight decrease in the tensile strength of the experimental steel. At 5-9 min, the distribution of C and Mn elements in the stabilization process of austenite has a major influence on the mechanical properties.

#### 3.3.2. Effect of Dual-Stable C-Mn Partitioning Process Time on Mechanical Properties

**Figure 11:**
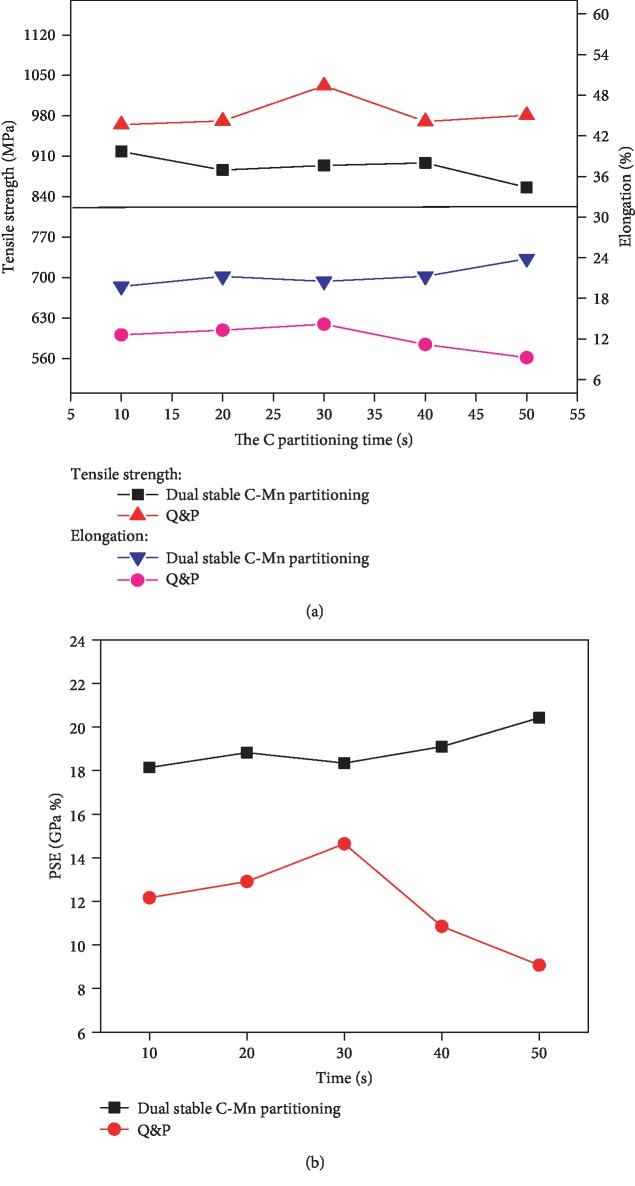
Mechanical properties of these steels after the dual-stable C-Mn partitioning process and Q&P process for 10 sec, 20 sec, 30 sec, 40 sec, and 50 sec: (a) strength and elongation and (b) product of strength and elongation.

In order to study the influence of the dual-stable C-Mn partitioning process time on the mechanical properties, after the first austenite stabilization of the tested steel is completed, it is quenched to 240°C and kept for 10-50 sec. The traditional Q&P process is carried out, that is, the tested steel is kept at 930°C for 5 min, then quenched to 240°C and kept for 10-50 sec.  Figure 11(a) shows the change of tensile strength and elongation with time in two processes. The tensile strength of dual-stable C-Mn partitioning process steel is lower than that of traditional Q&P process steel, and the elongation after fracture is obviously higher than Q&P process steel. The microstructure of the dual-stable C-Mn partitioning process steel is martensite, ferrite, and retained austenite. The ferrite produced by the first austenite stabilization process is a soft phase, and the volume fraction of the primary martensite of the tested steel is low [12, 23]. At the same time, during the first stabilization of the tested steel, C and Mn elements diffused from ferrite to austenite, the stability of austenite is increased, and finally, more retained austenite is obtained. In addition, the martensite formed first occurs self-tempering at the time of partition, and its plasticity is improved.

The tested steel under the Q&P process is kept at 930°C for 5 min, completely austenitized, and the C and Mn elements are evenly distributed in the austenite. The stability of austenite can only be enhanced by the C element at low temperature. During the partitioning process, carbon diffuses from martensite to untransformed austenite. Due to the low partition temperature, the carbon content of most untransformed austenite is less than the minimum carbon content required for no phase transition when cooling to room temperature. This is also the reason why the comprehensive mechanical properties of the double-stabilized C-Mn partitioning process are better than that of the traditional Q&P steel. The difference between the two products of strength and elongation is also less than 3.7GPa·%. The product of strength and elongation changes with time in the two processes. It can be seen from Figure 10(b) that after the dual-stable C-Mn partitioning process time is 30 sec, the product of strength and elongation of the dual-stable C-Mn partitioning process steel continues to increase with the increase of the holding time. The max product of strength and elongation is 20.42GPa·% after partitioning for 50 s. And the product of strength and elongation of traditional Q&P steel decreased sharply after the distribution time exceeded 30 s. This is due to the dynamic competition between the partitioning of C from martensite to retained austenite and the diffusion of C in austenite [12, 24]. At the same time, if the partitioning time is too long, some carbides will form, which will deteriorate the properties of steel. On the other hand, more austenite is retained in the partitioning of carbon and manganese. More austenite further enhances the product of strength and elongation. After the partitioning time exceeds 30 s, the product of strength and elongation begins to decrease. The reason for the decrease of the product of strength and elongation is the interface migration from martensite to austenite. The fine ferrite in the microstructure of the dual-stable C-Mn process steel increases the grain boundary density, hinders the diffusion of C element, and prolongs the time of C element dynamic balance. Macroscopically, the peak value of the product of strength and elongation is delayed.

## 4. Conclusion

1. The microstructure of the tested steel after the dual-stable C-Mn partitioning process is a multiphase structure of martensite, ferrite, and retained austenite. The martensite is lath-like shape, and the ferrite is flake-like shape. In this process, the C and Mn elements are enriched in austenite, and the stability of austenite is increased
2. With the increase of the first austenite stabilization time, the grain size of ferrite increases and the grain shape gradually changes from granular to flake, and the volume fraction increases; the tensile strength decreases gradually, and the elongation after fracture decreases first and then increases. The change rule of the product of strength and elongation is consistent with that of the elongation after fracture
3. As the second austenite stabilization time increases, the martensite volume fraction gradually decreases. After the second austenite stabilization time of 30 sec, the product of strength and elongation of the dual-stable C-Mn partitioning process steel continued to increase, while that of the traditional Q&P steel decreases, showing a delay of the peak value of the product of strength and elongation. Compared with traditional Q&P steel, the tensile strength of steel under the dual-stable C-Mn partitioning process is slightly lower, but the plasticity is obviously improved. The tensile strength is 875-910 MPa, the elongation after fracture is 20-24%, and the product of strength and elongation can reach 21GPa. %.

## Figures and Tables

**Table 1:** Main chemical composition of sample steel (wt. %).

Element	C	Mn	Si	Al	Cr	Ti	S	P	B
Content	0.11	1.50	1.16	0.043	0.024	0.01	0.001	0.008	0.003

**Table 2:** The fractions of ferrite for 10~50 sec.

Time (s)	10	20	30	40	50
Fraction (%)	46	36	49	62	56

## Data Availability

The all data used to support the findings of this study are included within the article.
